# Application of ^11^C-CHO in the imaging of cerebral alveolar echinococcosis: A prospective study

**DOI:** 10.1097/MD.0000000000042191

**Published:** 2025-05-23

**Authors:** Ameina Ayituhongman, Zhang Qizhou, Qin Yongde, Amina Yibulayin, Li Yubin, Li Xiaohong

**Affiliations:** a Department of Nuclear Medicine, The First Affiliated Hospital of Xinjiang Medical University, State Key Laboratory of Pathogenesis, Prevention and Treatment of High Incidence Diseases in Central Asia, Xinjiang Medical University, Urumqi, China.

**Keywords:** ^11^C-acetylcholine, ^18^F-FDG, cerebral alveolar echinococcosis

## Abstract

^11^C-acetylcholine (^11^C-CHO) is a positron emission tomography (PET) radiotracer that has been utilized in the diagnosis and treatment of prostate, esophageal, and lung cancers. This preliminary study aimed to assess the feasibility of using ^11^C-CHO PET/computed tomography (CT) for imaging cerebral alveolar echinococcosis (CAE). In a prospective single-center study, patients with CAE underwent ^18^F-fludeoxyglucose (^18^F-FDG) and ^11^C-CHO PET/CT imaging. The number and location of lesions detected by the 2 tracers were compared, along with determining the maximum standard uptake value (SUV_max_) of the lesions and the SUV_max_ values of the lesions/contralateral normal brain tissue (T/NT values). A total of 35 lesions (mean 4.3 lesions) in 8 CAE patients were imaged using both ^18^F-FDG and ^11^C-CHO PET/CT. The findings demonstrated that ^11^C-CHO PET/CT successfully detected all 35 lesions, achieving a 100% accuracy rate. This contrasted with the performance of ^18^F-FDG PET/CT, which only identified 24 lesions, resulting in an accuracy rate of 68.57%. Quantitative analysis further indicated that the SUV_max_ for ^11^C-CHO PET/CT averaged 3.13±0.93, compared to 9.35±2.84 for ^18^F-FDG PET/CT. The results of this study highlight the potential of ^11^C-CHO PET/CT as a novel imaging technique for CAE. In comparison to conventional ^18^F-FDG PET/CT, ^11^C-CHO PET/CT demonstrates superior accuracy in lesion detection, suggesting enhanced diagnostic capabilities and potential improvements in patient follow-up. Moving forward, conducting additional clinical studies will be crucial to corroborate and expand upon these promising findings.

## 1. Introduction

Alveolar echinococcosis (AE) is a zoonotic parasitic disease endemic to certain regions. The pathogen can disseminate through the bloodstream to various organs. When *Echinococcus multilocularis* larvae invade the brain, the condition is termed cerebral alveolar echinococcosis (CAE). CAE often presents with symptoms of increased intracranial pressure, such as headaches, papilledema, and vomiting, along with potential neurological deficits, significantly impacting the affected individuals’ quality of life.^[1]^

Surgery remains the primary treatment for CAE. However, the absence of a capsule around AE lesions complicates their differentiation from brain tissue. Consequently, relying solely on surgical observation and experience during excision poses risks. Incomplete removal can lead to recurrence, while excessive removal may cause vascular complications. Therefore, precise delineation of the lesion’s active borders is crucial.

For patients unsuitable for surgery, medication becomes a necessary treatment avenue. Albendazole liposomes, potent broad-spectrum antiparasitic drugs, effectively inhibit larval development by crossing the blood–brain barrier. However, in vitro studies on samples from patients undergoing long-term oral albendazole therapy suggest that lesions may retain biological activity. Hence, accurately assessing the active state of CAE and response to medication is imperative.^[2]^

Choline serves as a vital precursor for phospholipid synthesis, transported into cells via a high-affinity choline transporter and subsequently phosphorylated by choline kinase, incorporating it into phosphatidylcholine, a primary constituent of cell membrane phospholipids. Cells undergoing rapid proliferation exhibit heightened rates of membrane synthesis, correlating with increased choline uptake. Radiolabeled choline, such as ^11^C-acetylcholine (^11^C-CHO) or ^18^F-CHO, is employed in PET scans, notably in diagnosing and managing prostate, esophageal, and lung cancers. In normal brain tissue, choline uptake is relatively low. Given the limited reporting on ^11^C-CHO positron emission tomography/computed tomography (PET/CT) in diagnosing CAE, further research is warranted to evaluate its diagnostic efficacy in this context.

This preliminary study evaluated the feasibility of utilizing ^11^C-CHO PET/CT for imaging CAE by conducting paired scans with ^18^F-fludeoxyglucose (^18^F-FDG) and ^11^C-CHO.

## 2. Materials and methods

### 2.1. Study participants

This study enrolled patients with CAE who received treatment at the First Teaching Hospital of Xinjiang Medical University from January 2021 to January 2024. All participants met the inclusion and exclusion criteria and provided informed consent to participate in this study. This study conformed with the tenets of the Declaration of Helsinki and was approved by the First Affiliated Hospital of Xinjiang Medical University Clinical Research Ethics Committee.

### 2.2. Inclusion criteria

(1) Patients diagnosed with CAE based on clinical history, serological tests for echinococcosis, cranial MRI, and follow-up assessments; and (2) patients with pathological confirmation of CAE during follow-up.

### 2.3. Exclusion criteria

(1) Patients with other types of brain lesions; and (2) patients who were unable to cooperate or could not be followed up for diagnostic purposes.

### 2.4. PET/CT scan

All patients underwent both ^11^C-CHO and ^18^F-FDG PET/CT scans. ^11^C-CHO and ^18^F-FDG were produced by a GE MINItrace QILING cyclotron with a positron-emitting pharmaceutical production line and were synthesized via an automated module with a radiochemical purity of >95%. Images were acquired using the GE Discovery VCT PET/CT system at the Department of Nuclear Medicine, First Teaching Hospital of Xinjiang Medical University.

For ^18^F-FDG PET/CT, patients fasted for at least 6 hours, and their blood glucose levels were maintained below 7 mmol/L. Following this, they received an intravenous injection of ^18^F-FDG at a dosage of 7.4 MBq/kg. One hour after injection, a CT scan was conducted from the top of the skull to the lower cerebellum while the patient remained calm with their eyes closed. The CT parameters were as follows: voltage, 120 kV; tube current, 330 mA; collimator size, 64 × 0.625 mm; slice thickness, 2.5 mm; slice interval, 2.5 mm; rotation time, 0.6 ms; pitch, 0.983; and scan time, 5 to 10 seconds. Three-dimensional PET data collection occurred over the same range, capturing a one-bed position with a 5-minute acquisition time. Subsequently, the PET images were corrected for attenuation using CT data and reconstructed using the ordered-subsets expectation maximization algorithm to generate cross-sectional, coronal, sagittal, and PET/CT fusion images.

Within 1 week, patients also underwent ^11^C-CHO PET/CT scanning. This procedure did not require fasting. ^11^C-CHO was intravenously injected at the same dose of 7.4 MBq/kg. However, due to its short half-life of only 20.4 minutes, image acquisition was performed 5 to 10 minutes after injection. The scanning methods and parameters used for ^11^C-CHO PET/CT were consistent with those used for ^18^F-FDG PET/CT.

### 2.5. Image analysis

Brain PET/CT scans using ^18^F-FDG and ^11^C-CHO were conducted individually for 8 patients diagnosed with CAE. Three senior nuclear medicine physicians analyzed the resulting images and data on the same computer system using a blinded approach. This process involved measuring the standard uptake value (SUV)_max_ value within the region of interest of the lesion and independently evaluating and analyzing the imaging results for all cases. Conclusions were drawn based on the consensus of at least 2 of the 3 physicians. In the analysis of ^11^C-CHO PET/CT brain scans, the examination included measuring the SUV_max_ of the lesions and calculating the ratio of the SUV_max_ of the lesions to that of the contralateral normal brain tissue (T/NT ratio). For the ^18^F-FDG PET/CT brain scans, after accounting for normal physiological uptake, variations, and artifacts, the analysts scrutinized the scans for any areas showing abnormal ^18^F-FDG accumulation. They were then instructed to outline the biological boundaries of the CAE lesions, document the number and size of the lesions, and analyze the characteristics of the ^18^F-FDG distribution. Additionally, to delineate their characteristics, SUV_max_ values were measured for CAE lesions.

### 2.6. Statistical analysis

Statistical analysis was conducted using SPSS 21.0 software. Measurement data following a normal distribution are presented as the mean ± standard deviation (X ± S). Paired *t* tests were utilized to compare uptake values from 2 imaging agents for the same subject. Chi-square tests were employed to compare results between different imaging agents and to evaluate them against pathological outcomes. Furthermore, chi-square tests were performed to determine the positive rates and specificity of the ^18^F-FDG PET/CT and ^11^C-CHO PET/CT results.

## 3. Results

### 3.1. Clinical information

All 8 patients had a history of primary hepatic alveolar echinococcosis (HAE). MRI scans identified a total of 35 lesions, averaging 4.3 lesions per patient, with diameters ranging from 10 to 50 mm. All 8 patients presented with multiple lesions, predominantly located in the cerebral hemispheres (32 lesions), with 2 lesions in the cerebellum and one in the thalamus. Serum ELISA testing confirmed echinococcosis in 7 patients, while one tested negative.

Among the 8 patients, 2 underwent surgical treatment, during which 7 brain metastases were excised and subsequently confirmed by pathology to be AE. Following resection of HAE in 3 patients, no evidence of echinococcosis was observed elsewhere in the body, and ^18^FDG PET/CT scans revealed no significant metabolic activity in the brain lesions. Consequently, these patients displayed no clinical signs of increased intracranial pressure, and no further treatment was deemed necessary. The remaining 3 patients received drug therapy, including oral albendazole, in addition to dehydration therapy, steroids, and anti-seizure medications, as outlined in Table 1.

**Table 1:** Baseline characteristics of enrolled CAE patients.

Case	Sex	Age, Y	Number of lesions	Site	Serum hydatid ELISA	Type of treatment
1	M	48	4	Right frontal lobe, both occipital lobe	Positive	Drug
2	M	51	4	Left frontal lobe, both parietal lobe	Positive	None
3	F	53	3	Left cerebellum, left frontal lobe	Positive	Drug
4	M	42	7	Cerebellum, Left frontal lobe, left parietal lobe	Negative	Surgical
5	M	58	3	Right temporal lobe, right frontal lobe	Positive	None
6	M	47	4	Right parietal lobe, right frontal lobe	Positive	None
7	M	45	4	Left frontal lobe, left parietal lobe, Left cerebellum	Positive	Drug
8	F	37	6	Right parietal lobe, right frontal lobe	Negative	Surgical

### 3.2. ^18^F-FDG PET/CT imaging analysis

^18^F-FDG in CAE lesions showed varying degrees of uptake on PET/CT images. Seven CAE lesions with ^18^F-FDG uptake showing a concentrated blocky radioactive distribution; 10 lesions with ^18^F-FDG showing a ring-shaped concentrated radioactive distribution around the CAE lesions; 7 lesions with ^18^F-FDG showing radioactive distribution in CAE lesions with defects and sparse areas.

### 3.3. ^11^C-CHO PET/CT imaging analysis

**Figure 1.**
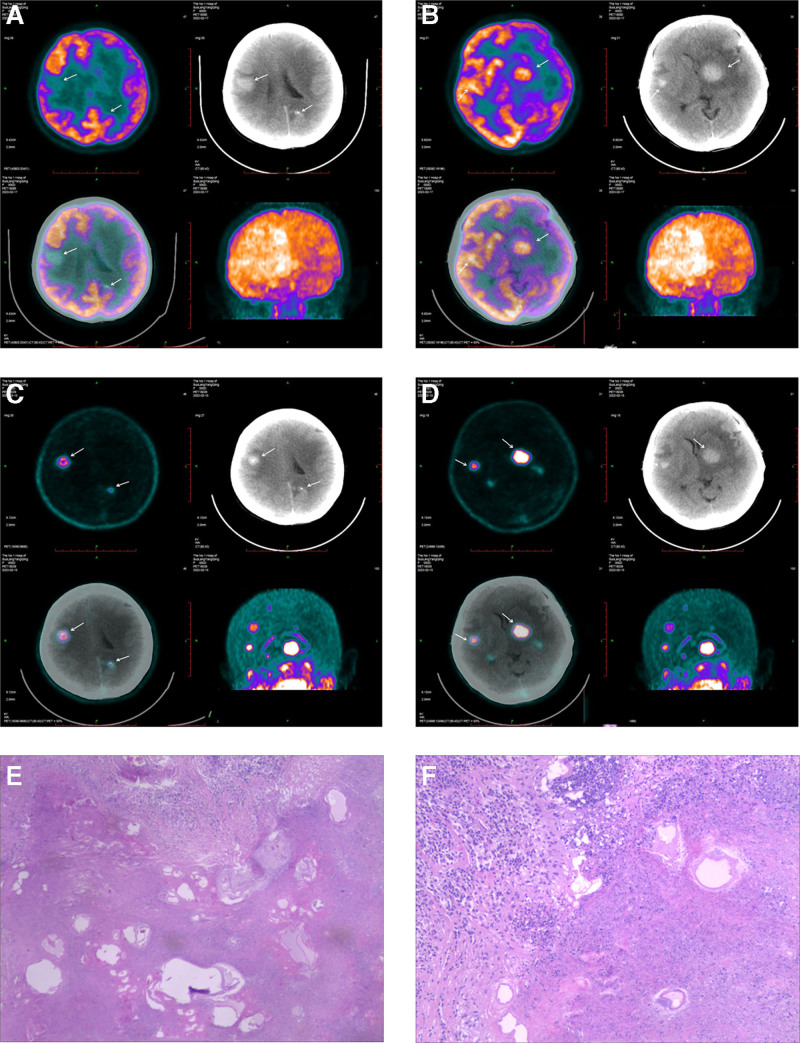
^18^F-FDG and ^11^C-CHO PET/CT imaging a 37-year-old female patient underwent brain ^18^F-FDG PET/CT imaging following a seizure episode. The scan revealed multiple mixed-density nodules in the brain. Some nodules exhibited significantly lower radioactive uptake than brain tissue, while others displayed uptake levels similar to brain tissue. The SUV_max_ was 14.1 (compared to the normal brain tissue SUV_max_ of 14.7), as depicted in A and B. Subsequently, the patient underwent brain ^11^C-CHO PET/CT imaging the following day, revealing multiple mixed-density nodules, all exhibiting higher radioactive uptake than the brain background. The SUV_max_ was 3.9 (compared to the normal brain tissue SUV_max_ of 0.2), as shown in C and D. Ultimately, the patient underwent surgical resection, and pathological examination confirmed CAE, as illustrated in E and F. ^18^F-FDG = ^18^F-fludeoxyglucose, PET/CT = positron emission tomography/computed tomography.

^11^C-CHO in CAE lesions showed varying degrees of uptake on PET/CT images. Thirteen CAE lesions with ^11^C-CHO uptake showing a concentrated blocky radioactive distribution; 11 lesions with ^11^C-CHO showing a ring-shaped concentrated radioactive distribution around the CAE lesions; 11 lesions with ^11^C-CHO uptake showing radioactive distribution in CAE lesions with defects and sparse areas (as showed in Fig. 1).

### 3.4. Comparison of ^11^C-CHO and ^18^F-FDG PET/CT imaging in patients with CAE

**Figure 2.**
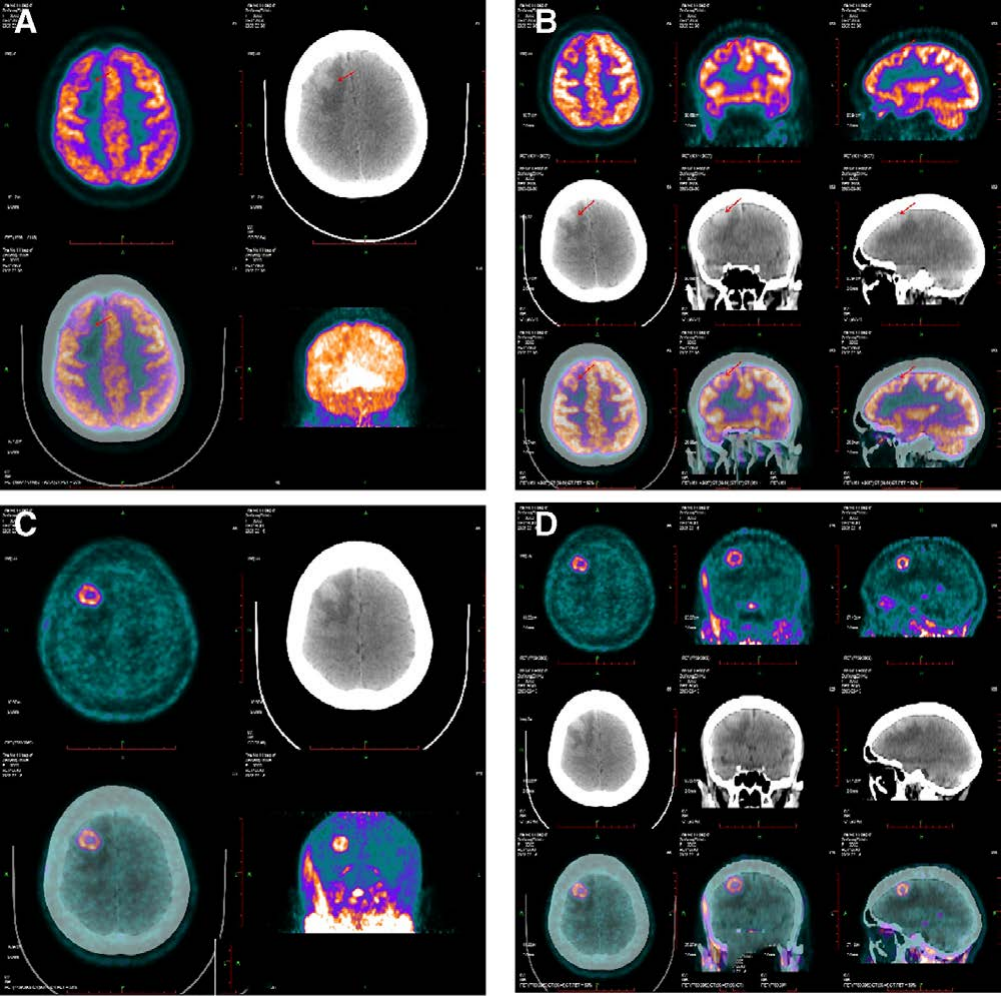
^18^F-FDG and ^11^C-CHO PET/CT imaging (A) ^18^F-FDG PET/CT imaging, (B) ^18^F-FDG PET/CT imaging, (C) ^11^C-CHO PET/CT imaging, (D)^11^C-CHO PET/CT imaging. ^18^F-FDG = ^18^F-fludeoxyglucose, PET/CT = positron emission tomography/computed tomography.

^11^C-CHO PET/CT and ^18^F-FDG PET/CT scans were conducted on 35 lesions in 8 patients diagnosed with CAE, as outlined in Table 2. The findings demonstrated that ^11^C-CHO PET/CT successfully detected all 35 lesions, achieving a 100% accuracy rate. This contrasted with the performance of ^18^F-FDG PET/CT, which only identified 24 lesions, resulting in an accuracy rate of 68.57%. Quantitative analysis further indicated that the SUV_max_ for ^11^C-CHO PET/CT averaged 3.13 ± 0.93, compared to 9.35 ± 2.84 for ^18^F-FDG PET/CT. The average size of the lesion was 3.198 × 2.631 cm on ^18^F-FDG, 2.615 × 2.234 cm on ^11^C-CHO, and 2.535 × 2.183 cm on MRI (as showed in Fig. 2).

**Table 2:** Diagnostic efficacy of ^11^C-CHO and ^18^F-FDG PET/CT imaging.

Radiotracer	Lesions	Accuracy rate	SUV_max_	Length of lesion (cm)	Width of lesion (cm)
^18^F-FDG	24/35	68.57%	9.35 ± 2.84	3.198 (2.283, 3.758)	2.631 (1.735, 3.5)
^11^C-CHO	35/35	100%	3.13 ± 0.93	2.615 (2.14, 3.403)	2.234 (1.533, 2.7)
MRI	35/35	100%	–	2.535 (2.091, 3.351)	2.183 (1.414, 2.531)

11 C-CHO** **= ^11^C-acetylcholine, ^18^F-FDG = ^18^F-fludeoxyglucose, PET/CT** **= positron emission tomography/computed tomography, SUV = standard uptake value.

## 4. Discussions

CAE lesions exhibit growth patterns similar to tumors. In their early stages, these lesions undergo granulomatous proliferative changes, with small *Echinococcus multilocularis* vesicles continuously infiltrating the surrounding tissue. As clusters of vesicles expand and proliferate, forming vesicle nests and scattered microvesicles, granulomas of varying sizes develop around them. Smaller granulomas typically consist of single nodules, whereas larger ones form through the fusion of several nodules, creating an invasive zone that can cause considerable harm. This process is indicative of the lesion’s potential to spread both inwardly and outwardly, similar to malignant tumors. As these vesicles grow outward, they can also affect nearby meninges. If proliferative lesions detach and spread through the cerebrospinal fluid, intracerebral metastasis can occur. The damage this disease causes to the host depends on its growth pattern and location, which resemble those of malignant tumors, both inward and outward.

Traditional imaging techniques such as CT and MRI play essential roles in assessing the therapeutic effects of albendazole in CAE treatment. Changes in lesion diameter represent a key indicator for evaluating treatment efficacy. Researchers have identified several ways to use CT and MRI to assess drug efficacy after treatment.^[3]^ These include (1) complete or nearly complete calcification of hepatic lesions (no significant changes in CT or MRI scans over 5 years), indicating patient recovery. This calcification can also indicate parasite death; (2) a decrease in lesion size with new irregular ring-shaped calcifications around the lesion, without low-density zones outside the calcification, suggesting improvement; (3) changes in the lesion’s edges from blurred to clear, while the size and shape remain consistent, indicating stability; and (4) growth of the lesion or the appearance of new lesions or distant metastases suggesting deterioration. However, while calcification can indicate reduced parasitic activity, it does not necessarily reflect the biological activity of the lesion itself. Calcification areas may coexist with active parasitic tissue, and completely inactive lesions might lack calcification. Therefore, relying solely on calcification to evaluate treatment efficacy can lead to misdiagnosis.^[4,5]^

One limitation of traditional imaging modalities, such as CT and MRI,^[6]^ is their inability to accurately assess the biological activity of CAE lesions. These diagnostic techniques rely on visual observations of lesion morphology, size, internal density, or signal variations, which often lack specificity. Consequently, they may fail to meet the follow-up demands of treated patients or assist clinicians in making informed decisions regarding the optimal time to discontinue treatment.

The uptake of the positron-emitting drug ^18^F-FDG utilized in this study is closely linked to cellular glucose metabolism. In chronic inflammation, the energy demands of specific inflammatory cells and proliferating fibroblasts increase, resulting in elevated glucose metabolism and varying degrees of increased ^18^F-FDG uptake. The level of inflammation correlates with the intensity of ^18^F-FDG uptake.^[7,8]^ The pathological features of the proliferative infiltration zone at the periphery of CAE lesions include a significant amount of intervesicular fibrous matrix, small vesicles, and various inflammatory cells such as eosinophils, lymphocytes, macrophages, and plasma cells. Some necrotic tissue edges are surrounded by fibrous tissue, with reactive glial cell proliferation in the adjacent area. These granulomatous lesions typically exhibit substantial ^18^F-FDG uptake, often forming a ring of intense concentration. Reuter et al employed PET to monitor ^18^F-FDG metabolism in HAE lesions, revealing that the lesion remained relatively stable when the edges of the liquefaction zone exhibited low radioactive uptake.^[9–11]^ However, a shift from low to localized high radioactive uptake at the edges of the lesion’s liquefaction zone indicated higher infiltration, suggesting that the “proliferative infiltration zone” or active lesion area serves as a critical indicator of disease progression. By dynamically monitoring the proliferative infiltration zone around CAE lesions to assess energy metabolism in active areas, the biological boundaries of the lesions can be accurately delineated for radical resection surgery. This approach aids in evaluating changes in activity following drug therapy, providing valuable insights to guide clinical management. To mitigate the issue of elevated background interference, our study combined ^18^F-FDG with ^11^C-CHO imaging, which offers a significant advantage in evaluating lesion activity. Choline, as a precursor to phosphatidylcholine, plays a crucial role in cell membrane synthesis, and ^11^C-CHO uptake reflects membrane synthesis activity in inflammatory cells, normal brain tissue SUV_max_ of 0.2, SUV value of ^11^C-CHO <1.5 can be considered as weak uptake.

Glucose serves as the primary energy source in the brain. Acting as a glucose analog, ^18^F-FDG is absorbed by normal brain cells, leading to uniform high-level metabolism. However, the pronounced radioactive uptake in the normal brain, particularly in the cortex, can obscure the ^18^F-FDG uptake at the peripheries of CAE lesions, posing challenges in determining whether these edges represent areas of proliferative infiltration. To address this limitation, our study utilized ^11^C-CHO imaging in combination with ^18^F-FDG, which significantly reduces the issue of elevated background interference. Choline found in the bloodstream acts as a precursor to phosphatidylcholine, a crucial constituent of cell membranes. The uptake rate of ^11^C-CHO by inflammatory cells mirrors the rate of membrane synthesis in tumor cells, offering a marker for assessing inflammatory cell activity. Furthermore, the proliferative infiltration zone surrounding CAE lesions contains numerous newly formed blood vessels and fibroblasts, and the proliferation of cell membranes in these vessels necessitates a substantial amount of choline. Consequently, in ^11^C-CHO imaging, the proliferative infiltration zone in CAE lesions may exhibit varying degrees of radioactive concentration. When the edge of CAE lesions demonstrates a circular dense zone of ^11^C-CHO uptake, it clearly reflects the biological activity of the lesion and its ability to infiltrate surrounding tissues. When the edge of CAE lesions shows sparse reduction or defect in the uptake of ^11^C-CHO, it indicates that the lesion is in a relatively stable phase and lacks the ability to infiltrate surrounding tissues. Absence of ^11^C-CHO uptake at the lesion edge indicates an inactive state. The significant advantage of ^11^C-CHO imaging is its minimal uptake in normal brain tissue, allowing for clearer visualization of lesions without interference from heightened background signals. This enables a more precise evaluation of the biological activity within the proliferative infiltration zone.

Therefore, ^11^C-CHO PET imaging provides a noninvasive, sensitive method to assess the activity of CAE lesions, overcoming the limitations of ^18^F-FDG in detecting active areas. By offering clearer delineation of active lesion areas, ^11^C-CHO PET can provide essential insights into treatment response and disease progression, making it a valuable tool for clinical monitoring.

## 5. Conclusion

The results of this study highlight the potential of ^11^C-CHO PET/CT as a novel imaging technique for CAE. In comparison to conventional ^18^F-FDG PET/CT, ^11^C-CHO PET/CT demonstrates superior accuracy in lesion detection, suggesting enhanced diagnostic capabilities and potential improvements in patient follow-up. Moving forward, conducting additional clinical studies will be crucial to corroborate and expand upon these promising findings.

## Author contributions

**Data curation:** Qin Yongde.

**Funding acquisition:** Li Xiaohong.

**Methodology:** Ameina Ayituhongman, Zhang Qizhou, Amina yibulayin, Li Yubin, Li Xiaohong.

**Project administration:** Qin Yongde, Li Yubin.

**Writing – original draft:** Ameina Ayituhongman, Li Xiaohong.

**Writing – review & editing:** Qin Yongde, Li Xiaohong.
